# Consumer knowledge and practices to pork safety in two *Taenia solium* cysticercosis endemic districts in Eastern Cape Province of South Africa

**DOI:** 10.1186/s12879-020-4839-9

**Published:** 2020-02-06

**Authors:** Msawenkosi I. Sithole, Johan L. Bekker, Samson Mukaratirwa

**Affiliations:** 1 Department of Environmental Health, Tshwane University of Technology, Pretoria, 0001 South Africa; 2 School of Life Sciences, University of KwaZulu-Natal, Westville Campus, Durban, South Africa

**Keywords:** *Taenia solium* cysticercosis, Food/meat safety, Consumer knowledge and practices

## Abstract

**Background:**

Globally, *Taenia solium* can cause cysticercosis in humans (including neurocysticercosis) and in pigs through ingestion of eggs and taeniasis in humans through ingestion of raw/undercooked pork contaminated with mature cysts. It is now recognised globally as one of the most prevalent food-borne parasitic diseases. The majority of cases have been reported in developing countries where consumption of food produced under unhygienic conditions is prevalent, exacerbated by lack of food safety education. The aim of this study was to determine the knowledge and practices of consumers towards pork safety in two districts of the Eastern Cape Province of South Africa, where *T. solium* cysticercosis is endemic in pigs and humans.

**Methods:**

Three-hundred-and-sixty-one (361) participants were conveniently interviewed on consumer knowledge (harmfulness of *T. solium* cysticercosis, ability to identify cysts, trustworthiness of registered butcheries and legal requirements) and practices (storage of pork and method(s) of cooking pork safely) through a structured questionnaire. Chi-square for association of variables was used to compare differences in the districts.

**Results:**

Overall, 73.1% of the study group from both districts agreed that pork forms an important part of their diet. Consumers (54.2%: 189/349) agreed that pork infected with *T. solium* cysts could be harmful, and 57.3% (188/328) indicated their inability to identify *T. solium* cysts in pork when slaughtered at home. Although 69.5% (234/352) trusted pork bought from butcheries, only 52.2% (187/358) were aware that butcheries must present a registration certificate in order to operate. This coincides with the fact that very few (< 10%) were aware of the legal requirements in terms of disease control, slaughter and food preparation. Most consumers (88.7%: 268/302) kept pork in the fridge and only 11.3% (34/302) kept it in a freezer (*p* = 0.02). Although not significantly different between the districts (*p* = 0.15), consumers in Alfred Nzo (71.4%: 152/213) and OR Tambo (61.2%: 74/12) mostly cooked pork as a stew, followed by braai/barbeque and frying or baking. This was in line with the fact that consumers in Alfred Nzo (79%: 147/186) and OR Tambo (80.8%: 120) preferred well-cooked pork; the main reason for this was the belief that cooking kills germs (43.6%: 121/277) followed by rendering the meat tasty (26.4%: 73/277).

**Conclusions:**

Consumers surveyed in the two districts were somewhat aware that *T. solium* cysticercosis could be harmful, although some were not able to identify *T. solium* cysts in pork. They also lacked sufficient knowledge regarding butchery certification and other legal requirements related to disease control, slaughter and food preparation. Practices related to cooking have the potential to promote the transmission of human taeniasis and the fact that most respondents preferred stewed pork could be a positive sign, as the cysts are destroyed during the cooking process. Results from this study are useful for the development of a control and prevention strategy targeted towards consumers, and the creation of awareness of food safety, with special emphasis on *T, solium* cysticercosis.

## Background

Food-borne diseases have become widespread and are a serious public health problem, especially in industrialised countries where the percentage of people infected by food-borne diseases each year has been increasing [1]. This has been attributed to industrialisation of animal production, mass food processing and distribution, globalisation of the food trade and increased mobility of people and goods around the world [1, 2]. Food-borne parasitic diseases result in considerable morbidity and mortality [3], and *T. solium* is among the list of food-borne zoonotic parasites known to cause epilepsy and other neurological sequelae in populations across sub-Saharan Africa [3]. When pig (intermediate host) ingests faeces contaminated with *T. solium* eggs, larvae invade the body of the animal, mostly in the subcutaneous fat, muscles or brain, resulting in porcine cysticercosis. When human (definitive host) ingests infected (metacestode) in under-cooked or raw pork this develops to mature form of the tapeworm, causing taeniasis. Humans occasionally become intermediate hosts by drinking water or eating food contaminated with *T. solium* eggs or by transferring eggs to mouth with contaminated hands or through auto-infection. This may result in human cysticercosis and/or neurocysticercosis. *Taenia solium* cysticercosis is an underreported and neglected zoonotic disease in man and pigs in many developing countries [4] and has been classified as one of the most important global foodborne parasites [5]. The public health and economic impacts are difficult to determine because of the complexities of the disease in resource-poor livestock farming and the economic loss due to meat condemnation (lack of affordable diagnostic tools in humans, ineffectiveness of meat inspection in detecting cysts, home slaughter of pigs and stigma related to NCC in endemic areas) [6–8]. In South Africa, the diseases’ cost was estimated to be USD 5 million for the agricultural sector alone, with an overall cost of between USD 18.6 million and USD 34.2 million [6].

Despite strong evidence indicating that *T. solium* is an important pathogen of resource-poor, pig farming communities in South Africa, there has been only one report on the prevalence of porcine cysticercosis of 64.6% in the villages of Alfred Nzo and OR Tambo Districts of the Eastern Cape Province, South Africa [9]. However in the past 30 years, several hospital surveys utilising serological and/or radiological diagnostic techniques have indicated that 28–50% of epileptic cases including children were positive for cysticercosis [10]. Inactive and active NCC detected through computed tomography CT scans were found in 61.1% of patients with epilepsy in Lusikisiki, in the Alfred Nzo District of South Africa [11].

South Africa has the largest number of pigs in sub-Saharan Africa, of which approximately 25% are free ranging and owned by emerging pig producers in resource-poor areas of the country [9, 12]. As part of this study, Sithole et al. [13] reported on pig husbandry practices, pig health management, feeding, marketing and slaughter of pigs by farmers in two districts of Eastern Cape Province, South Africa. Results showed that pigs were managed under traditional free range, resulting in them roaming freely with other domestic animals such as cattle, sheep, goats, chickens, dogs and game.

Although meat inspection has traditionally been the main means of control to detect cysts at slaughter, the effectiveness of the method is poor and has some limitations in terms of sensitivity [14, 15]. Sithole et al. [16] also confirmed that meat inspection alone was insufficient to detect porcine cysticercosis and therefore suggested additional multiple incisions on the heart, tongue, shank and masticatory muscles to improve cyst detection.

Although studies on *T. solium* cysticercosis have been conducted in Alfred Nzo and OR Tambo Districts [12, 17] no study has been carried out on consumer knowledge and practices on pork safety. The objective of this study therefore was to assess consumer knowledge and practices of pork safety in two *T. solium* cysticercosis endemic districts in Eastern Cape Province, South Africa.

## Methods

### Study areas

The study was conducted in villages located in two district municipalities of Eastern Cape Province, namely Alfred Nzo and O.R. Tambo Districts and the demographic characteristics and prevalence of *T. solium* taeniasis/cysticerosis in the two locations have been previously described [17].

### Study design

This study forms part of a larger study related to the occurrence and detection of *T. solium* in Eastern Cape Province of South Africa. A cross-sectional community-based study was conducted between August and October 2016. Prior to commencement of the survey, an inception meeting was held with the representatives of the local House of Traditional Leaders to explain the purpose of the study. Convenience sampling, which is a non-probability sampling technique, was used to select respondents based on their accessibility and willingness to participate in the study.

Three-hundred-and-sixty-one (361) household heads, originating from 30 villages of Alfred Nzo (*n* = 236) and OR Tambo (*n* = 125) districts were included. A structured questionnaire collected information from consumers on demographics (age, gender, level of education), consumer knowledge (knowledge of *T. solium* cysticercosis, ability to identify cysts, trustworthiness of butcheries and legal requirements) and practices (storage, methods of cooking) towards pork safety. The questionnaire was translated into IsiXhosa, which is the native language of both the study districts and was pre-tested with five consumers per district and improvements were made prior to the administration to study participants. The pre-tested questionnaires were not included in the results. Due to the high level of illiteracy among villagers, interviews, with the aid of the translated structured questionnaire, were conducted by the researcher and a research assistant both of whom were conversant with IsiXhosa. In South Africa, persons under 18 years of age are regarded as minors therefore, no such person was interviewed during the study.

### Data analysis

Due to the nature of the questions, not all were answered by respondents and therefore the sample size (n) of the variables differed. Frequencies observed within the categories of each question and between districts were tested by constructing row x column frequency tables of meaningful associations and a Chi-square (χ^2^) test for independence (association) of variables (*p* ≤ 0.05 at 95% CL were regarded as significantly different) [18]. All data analyses were done using SAS Statistical Software [19].

## Results

In total, 361 questionnaires were completed, of which 65.4% (236) were from Alfred Nzo and 34.6% (125) from OR Tambo district (*p* = 0.03). Overall, 73.1% of the respondents from the two districts confirmed that pork formed an important part of their diet.

### Socio-demographic information of the study districts

Table 1 shows the socio-demographic characteristics of respondents from the two study districts. Gender distribution between the two districts differed significantly (*p* = 0.03), with most respondents being males (56.2%: 203/361). Of the 296 (*n* = 358) from both districts who indicated that they purchase meat themselves, 56% were males. The age distribution of respondents from the two districts was significantly different (χ^2^_(df = 5)_ 50.0; *p* = 0.01), with a mean age of 45 years (range = 18–88 years) for Alfred Nzo, and 34 years (range = 18–80 years) for OR Tambo. The number of family members per household for both districts ranged from one to five, with a mean of three (χ^2^_(df = 4)_ 7.3; *p* = 0.11). A higher rate of unemployment (χ^2^_(df = 4)_ 31.6; *p* = 0.01) was observed in OR Tambo (47.2%: 58/123) than in Alfred Nzo (29.8%: 70/235). A high number of respondents in both Alfred Nzo (50.0%: 118/236) and OR Tambo (43.2%: 54/125) had an education (χ^2^_(df = 3)_ 4.7; *p* = 0.19) below grade 9.

**Table 1:** Demographic characteristics of respondents on pork safety from Alfred Nzo and OR Tambo Districts in Eastern Cape Province of South Africa

Factor	Number of respondents	Alfred Nzo District (*n* = 236)	OR Tambo District (*n* = 125)	χ^2^	*P*-value
Gender	361			4.69	0.03
Male		123 (52.1%)	80 (64.0%)		
Female		113 (47.9%)	45 (36.0%)		
Age Group	361			50.00	0.01
≤ 24		19 (8.1%)	36 (28.8%)		
25–34		48 (20.3%)	31 (24.8%)		
35–44		43 (18.2%)	21 (16.8%)		
45–54		56 (23.7%)	23 (18.4%)		
55–64		37 (15.7%)	9 (7.2%)		
> 64		33 (14.0%)	5 (4.0%)		
Mean		45 years	34 years		
Members in a household	361			7.33	0.12
≤ 2		14 (6.0%)	10 (8.0%)		
3–5		84 (35.6%)	30 (24.0%)		
6–8		83 (35.2%)	56 (44.8%)		
9–11		32 (13.6%)	13 (10.4%)		
> 11		23 (9.8%)	16 (12.8%)		
Mean		3	3		
Source of income	358			31.59	0.01
Work		41 (17.5%)	21 (17.1%)		
Self-employed		72 (30.6%)	11 (8.9%)		
Grants		24 (10.2%)	25 (20.3%)		
Pension		28 (11.9%)	8 (6.5%)		
Unemployed		70 (29.8%)	58 (47.2%)		
Education	361			4.72	0.19
No education		22 (9.3%)	9 (7.2%)		
Grades 1–8		118 (50.0%)	54 (43.2%)		
Grades 9–12		90 (38.1%)	61 (48.8%)		
Tertiary level		6 (2.5%)	1 (0.8%)		

Note: *p* < 0.05 denote significant difference between the Alfred Nzo and OR Tambo Districts

### Consumer knowledge

Table 2 shows 54.2% (*n* = 349) of the consumers in Alfred Nzo (*n* = 110/228) and OR Tambo (*n* = 79/121) (*p* = 0.01) expressed that pork with cysticercosis may be harmful to their health. Furthermore, 42.7% (*n* = 328) in Alfred Nzo (95/211) and OR Tambo (45/117) indicated they could identify cysts when they slaughtered pigs at home, and in IsiXhosa (native language) referred to it as “Amaqhakuva, Amaqhanqa or Intshulube”. It was unclear if they knew what measly pork looked like, as many respondents verbally indicated they could not distinguish between a piece of fat or a cyst. Majority of consumers were not aware of the symptoms related to human cysticercosis. In Alfred Nzo, 39.8% (140/227) trusted pork purchased from local butcheries compared to OR Tambo, where only 26.7% (94/125) trusted such pork (*p* = 0.01). In Alfred Nzo, 68.6% (131/191) of respondents and 70.9% (88/124) from O.R. Tambo believed butcheries were inspected on a regular basis for food hygiene (*p* = 0.65). Only 48.2% (113/234) in Alfred Nzo and 59.7% (74/124) in OR Tambo were aware that butcheries must have a registration certificate (*p* = 0.04) in order to operate. Generally, consumers lacked knowledge regarding existing meat/food safety legislations as they are not aware of the existence of legislation, such as the Animal Disease Act (90.8%: 277/305) (*p* = 0.01), Meat Safety Act (90.0%: 276/307) (*p* = 0.01), Foodstuff, Cosmetics and Disinfectant Act (91.1%: 266/292) (*p* = 0.01) and National Health Act (89.5%: 273/305) (*p* = 0.01).

**Table 2:** Consumer knowledge towards pork infected with *Taenia solium* cysticercosis in two districts in Eastern Cape Province, South Africa

Factor	Total respondents	Alfred Nzo District		OR Tambo District		Total Yes %	Total No %	χ^2^	*P*-value
		Yes	No	Yes	No				
Knowledge									
Do you think pork with cysticercosis may be harmful to consumers?	349	110	118	79	42	54.2	45.8	9.24	0.01
Are you aware of human symptoms associated with *Taenia solium* cysticercosis?									
i. Headache	315	11	203	18	83	11.5	18.5	13.20	0.01
ii. Confusion	316	3	212	15	86	8.1	91.9	23.16	<.01
iii. Seizure	315	15	200	25	75	16.0	84.0	19.99	<.01
iv. Change vision	316	3	212	11	90	4.4	95.6	14.63	0.01
v. Imbalance	319	7	211	13	88	6.3	93.7	10.96	0.01
vi. Death	318	9	207	21	81	9.4	90.6	21.86	< 0.01
Can you identify cysts in pork?	328	95	116	45	72	42.7	57.3	1.32	0.25
Have you received training on cysts identification in pork?	357	2	231	6	118	2.24	97.75	5.85	0.01
Do you trust pork that you buy from the butchery?	352	140	87	94	31	66.5	33.5	6.61	0.01
Do you know that butcheries are inspected on regular basis for food hygiene?	315	131	60	88	36	69.5	30.5	0.20	0.65
Are you aware that butcheries must present a registration certificate?	358	113	121	74	50	52.2	47.8	4.21	0.01
Are you aware of the following legislations:									
i Animal Disease Act	305	5	177	23	100	9.2	90.8	22.40	0.01
ii. Meat Safety Act	307	6	177	25	99	10.0	90.0	23.20	0.01
iii. Foodstuffs, Cosmetics and Disinfectant Act	292	2	167	24	99	8.9	91.1	29.48	0.01
iv. National Health Act	305	6	176	26	97	10.5	89.5	24.87	0.01

Note: *p* < 0.05 denote significant difference in association between the Alfred Nzo and OR Tambo Districts

### Consumer practices

A summary of the respondents’ practices on pork preparations and consumptions is shown in Table 3. Most respondents in the two districts (88.7%: 268/302) stored their pork in a fridge, while only a small number (11.3%: 34/302) used freezers (χ^2^_**(df = 1)**_ 5.0; *p* = 0.05). The majority of respondents from both districts (67.7%: 226/334) preferred the traditional cooking method of stew compared to braai (barbecue) (30.2%: 95/334), frying (2.5%: 9/334) and baking (1%: 4/334) (χ^2^_**(df = 4)**_ 6.7; *p* = 0.15). Most respondents of the two districts (79.9%: 244/306) who preferred pork to be cooked well (well done) (χ^2^_**(df = 3)**_ 2.6; *p* = 0.45); 42.9% (121/277) indicated the main reason for “well done” cooking was that it killed germs (χ^2^_**(df = 4)**_ 7.5; *p* = 0.11).

**Table 3:** Consumer practices in cooking and consumption of pork in two districts endemic for *Taenia solium* cysticercosis in Eastern Cape Province, South Africa

Factor	Number of respondents	Alfred Nzo District	OR Tambo District	χ^2^	*P*-value
Where do you keep your pork after slaughter/buying?	302			5.03	0.05
Fridge		151 (85.3%)	117 (93.6%)		
Freezer		26 (14.7%)	8 (6.4%)		
How do you cook your pork?	334			6.72	0.15
Stew		152 (71.4%)	74 (61.2%)		
Braai		51 (23.9%)	44 (36.4%)		
Fry in oil or fat		7 (3.3%)	2 (1.7%)		
Bake in oven		2 (0.9%)	1 (0.7%)		
Other (unspecified)		1 (0.5%)	0 (0.0%)		
How do you prefer to eat your pork?	306			2.62	0.45
Well done		147 (79%)	97 (80.8%)		
Medium to well-done		27 (14.5%)	15 (12.5%)		
Medium to rare		9 (4.9%)	8 (6.7%)		
Rare		3 (1.6%)	0 (0.0%)		
What is the reason you want your pork to be cooked this way?	277			7.54	0.11
Kill gems		77 (47.9%)	44 (37.9%)		
Tasty		42 (26.1%)	31 (26.8%)		
Stop blood		21 (13%)	28 (24.1%)		
Soft		19 (11.8%)	10 (8.6%)		
Tender		2 (1.2%)	3 (2.6%)		

Note: *p* < 0.05 denote significant difference in association between the Alfred Nzo and OR Tambo Districts

## Discussion

There is a strong relationship between consumer perception of risks, trust in food safety and purchasing behaviour [20]. This study revealed that there were more men responsible for meat purchasing compared to women, which was an interesting phenomenon. In most African cultural practices, it is the responsibility of women to buy and cook food for the family [21], as women tend to be more aware of food safety than men [22]. In this case, a possible intervention would be to establish educational food safety programmes specifically for food handlers. The high level of unemployment was comparable to that of previous studies (approximately 55%) in the area [9, 23] and this compels people to buy pork and other meat from cheap informal/illegal food vendors, or to slaughter animals themselves for own consumption and commercial purposes to enhance income [24]. Of concern was that a high percentage of consumers had an education level below grade 9, which is the minimum school education level in South Africa [25]. This result is similar to that of other reports in Alfred Nzo, where 58% of respondents were without formal education [26] and OR Tambo had 26% with no schooling at all [27].

It has been reported that a low level of education exacerbates a high unemployment rate [25, 28, 29], and this may impact negatively on public health initiatives targeted to improve consumers knowledge and practices on consumption of safe pork [30]. Due to the low level of education, the general lack of knowledge on food legislation was expected. Improving the knowledge levels of consumers could advance food safety legislation awareness [31, 32]. In the South African context, awareness regarding hygiene practices and legal requirements may be enhanced by structures such as National and Provincial Departments of Health and of Agriculture, Food and Fisheries and the National Consumer Commission of South Africa who are mandated to ensure the welfare of consumers, which includes the implementation of relevant policies and strategies to ensure safe pork production in rural settings, such as the areas from this study.

Even though participants referred to cysts as “Amaqhakuva, Amaqhanqa or Intshulube” in IsiXhosa, it was of particular concern that almost half of the participants (45.9%) believed that pork with cysticercosis may not be harmful. This could be ascribed to the complexity of the life cycle of *T. solium* involving humans, pigs and environment; lack of knowledge and awareness of the public health importance of the parasite. Furthermore, since pork was bought from local butcheries there might be an element of trust that the pork was safe, without realising the possible consequences of consuming uninspected pork infected with *T. solium* cysts. This was similar to studies in Tanzania and Zimbabwe [33, 34], where it was reported that infected pork was purchased from unregistered fresh meat markets. A study conducted by Soji et al. [35], in South Africa, reported that consumers do not mind purchasing meat from formal or informal suppliers as long as it is affordable. This constitutes the need for consumer awareness campaigns by relevant authorities and other stakeholders. The development of schemes that would allow for gradual development of compliance would allow for interim registration and monitoring of butcheries on a regular basis to ensure safe meat products for human consumption (Powell et al., 2013).

The low percentage of consumers using freezers may be ascribed to the unaffordability thereof and the unavailability of electricity in most villages. This is an unfortunate situation as freezing of meat with *T. solium* cysts at − 5 °C to -24 °C for 1 to 4 days can inactivate *T. solium* cysts [36]. The South African red meat regulations prescribe temperatures of − 10 °C for 10 days or − 18 °C for 3 days to inactivate the *T. solium* in pork meat [37]. It was proven that domestic freezers were capable of maintaining temperatures of − 20.3 °C, [38], which fell within the aforementioned temperature ranges and thus should have the capacity to deactivate *T. solium* cysts. However, the fact that most consumers preferred stewed pork was fortunate as this normally requires slow cooking for longer periods [39] wherein *T. solium* cysts can be inactivated when the pork reach an internal temperature of 65 °C [40]. The other preparation methods, such as braai/barbeque, especially medium to rare are risky, as *T. solium* cysts may not be killed and consumers are exposed to infection [41].

## Conclusion

This study confirmed that the majority of consumers from the study areas believed pork formed part of their diet as a source of protein, thus emphasising that rural pig farming would remain a source of livelihood for resource-poor farmers. Ensuring food safety, requires effective food safety systems that are vital to maintain consumer confidence and provide a sound regulatory foundation for domestic and international trade in food, which supports economic development [42]. This study revealed that pork consumers in the study areas have the likelihood of being exposed to pork infected with *T. solium* cysts, mainly due to eating uninspected pork derived from home-slaughtered pigs or buying of pork from unregistered sources and following unsafe cooking practices.

Given the importance of this parasite, awareness programmes for consumers should be initiated on the importance of consuming safe pork, and the zoonotic importance of *T. solium* since the parasite is endemic in these areas.

## Data Availability

The data sets analysed during the current study are available from the corresponding author on reasonable request.

## Abbreviations

CT: Computed tomography
NCC: Neurocysticercosis
OR Tambo: Oliver Reginald Tambo
SAS: Statistical analysis system
*T. solium*: *Taenia solium*
